# Treatment of Oesophagojejunostomy Leakage With the Use of Fibrin Glue: Case Report

**DOI:** 10.7759/cureus.21573

**Published:** 2022-01-24

**Authors:** Władysław Skałba, Piotr Szymański, Marek Czarnecki, Marcin Zeman

**Affiliations:** 1 Department of Oncological and Reconstructive Surgery, Maria Sklodowska-Curie National Research Institute of Oncology, Gliwice, POL

**Keywords:** endoscopic treatment, sems, oesophageal stents, fibrin glue, gastric cancer, gastrectomy, anastomotic leakage, oesophagojejunostomy leakage

## Abstract

Oesophagojejunostomy leakage after total gastrectomy with D2 lymphadenectomy remains a significant clinical issue. In this paper, we present a case of a 63-year-old female patient who, on the first day after surgery, was diagnosed with oesophagojejunostomy leakage in the chest. The general condition of the patient was stabilized by the implementation of conservative treatment and thoracic drainage. Thanks to covered oesophageal stents, the leakage from the fistula between the anastomotic connection, pleura, and skin was reduced. In the subsequent step, treatment with fibrin glue resulted in complete closure of the fistula. The complementary use of fibrin glue may be effective in the treatment of small oesophagojejunostomy leakages when other endoscopic methods are not sufficient.

## Introduction

Currently, the fundamental part of the combined treatment of gastric cancer is a surgical procedure. According to current recommendations, cancer staging higher than T1b is an indication of the performance of total or partial gastrectomy with D2 lymphadenectomy [1]. Despite continuous development of postoperative management, including antibiotic therapy, nutritional treatment, and rehabilitation, there are still cases of postoperative complications. Oesophagojejunostomy leakage is one of the most serious complications after gastrectomy. It is estimated that it may affect between 4% and 9% of patients after surgical treatment [2-3]. However, mortality resulting from leakage may reach even 32.5% [4]. With the constant development of minimally invasive and endoscopic techniques, an ever-higher percentage of anastomotic leakages are successfully treated with these methods [5-17].

## Case presentation

**Figure 1:**
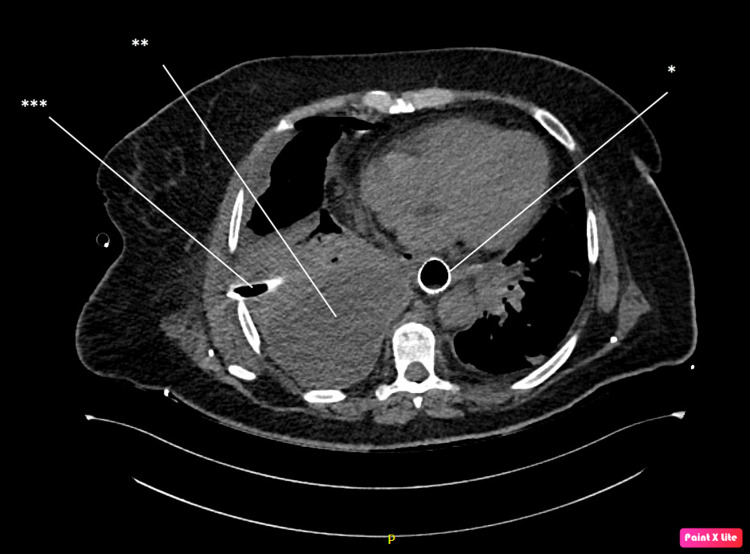
Computer tomography with oral administration of a contrast agent, post stent implantation (*). No release of contrast beyond the gastrointestinal lumen is seen. Pleural empyema (**) with a drain inside (***) is seen.

A 63-year-old patient, with a diagnosis of stomach cancer, after the administration of four cycles of preoperative chemotherapy, was admitted to the department of oncological and reconstructive surgery for surgical treatment. The patient did not report weight loss three months before surgery, she had a Nutritional Risk Screening (NRS) score of 2. She was qualified for surgical treatment in the American Society of Anaesthesiology (ASA) 2 category due to comorbidities. Intraoperatively, the presence of a tumor was confirmed in the subcardial region, reaching the lesser curvature (Siewert type III). A cytologic examination of peritoneal washings showed no presence of neoplastic cells. Total gastrectomy with D2 lymphadenectomy and Roux-en-Y loop reconstruction with manual oesophagojejunostomy using single-layer PDS 3-0 suture was performed. An air leak test revealed slight leakage in the right lateral wall, which was treated by the application of another suture. Another air leak test was negative. Two abdominal drains were kept. Because the first air leak test was positive, on the first day after surgery control endoscopy was performed. At the right side of anastomosis, a 5 mm wide concentration of fibrin was found, which led to suspicion of oesophagojejunostomy leakage. The 11 cm covered oesophageal stent was placed in the anastomotic connection area using a C-arm-guided approach. Total parenteral nutrition and antibiotic therapy were implemented. Computed tomography (CT) scan revealed a presence of fluid in both pleural cavities, so bilateral drainage was performed resulting in an outflow of pus, which was collected for culture. On the seventh day after surgery, a CT scan showed no leakage, but it revealed a presence of an encapsulated pus cistern in the right pleural cavity, which was treated with ultrasound-guided drainage. See Figure 1.

**Figure 2:**
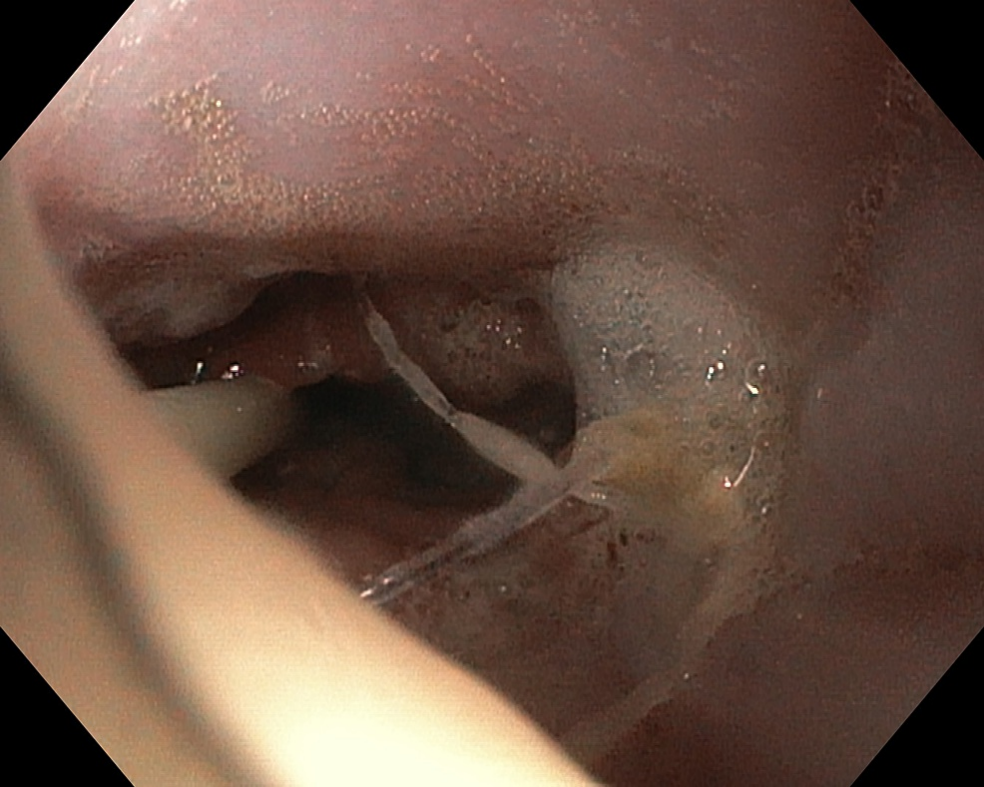
Endoscopic image of the anastomosis area with the site of leakage visualized with the use of hydrogen peroxide

After treatment, the patient's condition gradually improved, the pain was reduced, and the patient was fully mobilized. The values of inflammatory parameters, including C-reactive protein, were reduced (from 420 mg/l to 273 mg/l). On day 10 after surgery, the patient was given an oral solution of methylene blue, revealing leakage between the gastrointestinal tract and the right pleural cavity. Control endoscopy was performed and the oesophageal prosthesis was removed. The anastomosis showed no evident signs of leakage but due to the course of the disease, a decision was made to re-implant an 11 cm covered oesophageal prosthesis. Further decrease in the inflammatory parameter levels was observed, but the pus drainage from the right pleural cavity was maintained, although got lowered to 150 ml per 24 hours. Another test with oral administration of methylene blue confirmed persisting leakage. Therefore, on day 44 after surgery, another attempt at endoscopic treatment was made. Under general anesthesia, a gastroscope was inserted in the oesophagojejunostomy area. No evident signs of leakage were found. A solution of hydrogen peroxide was administered through the drain in the right pleural cavity, whereby the fistula canal with a diameter of 2-3 mm was visualized in the anastomosis. See Figure 2.

**Figure 3:**
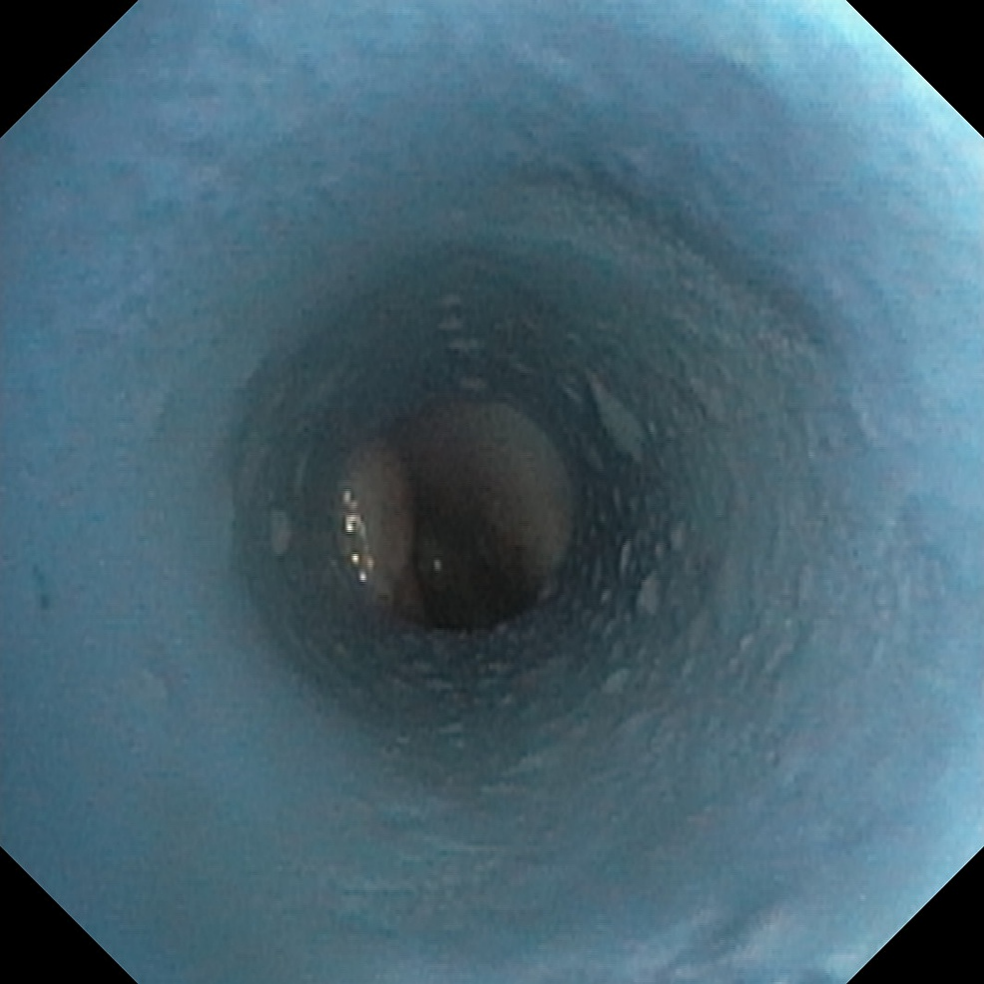
Endoscopic image obtained after the insertion of a bronchoscope in the drain placed inside pleural empyema. The fistula opening is seen in the background.

A bronchoscope was introduced via the drain in the pleura. The fistula opening was visualized directly in front of the tip. See Figure 3.

**Figure 4:**
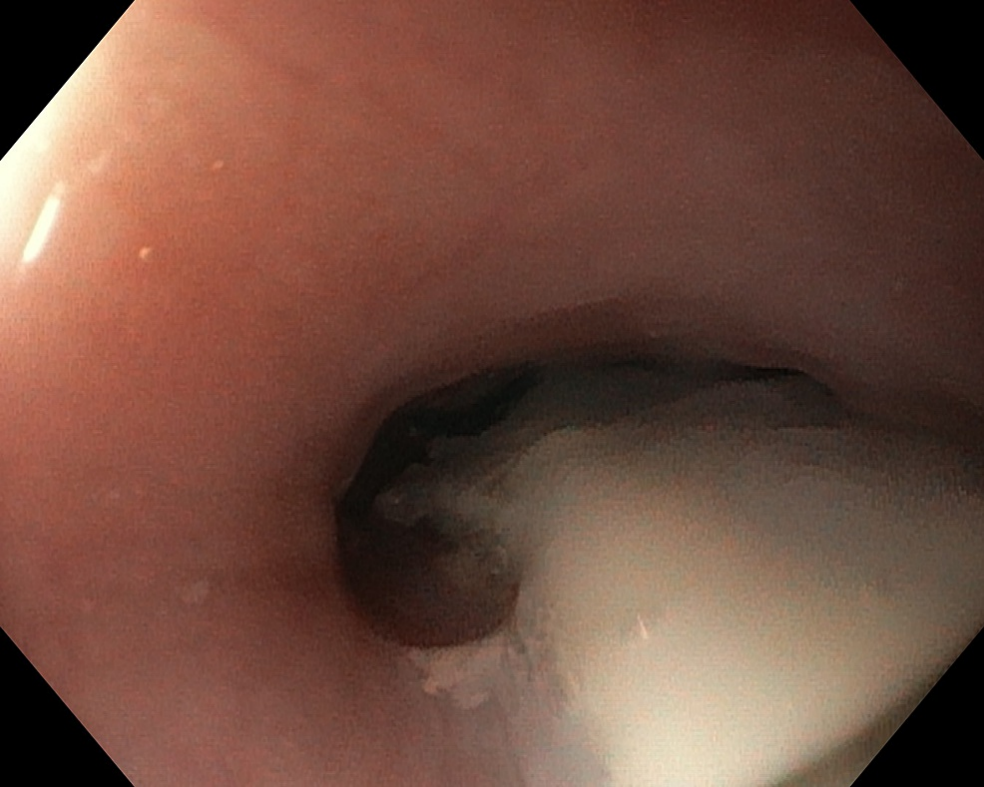
Endoscopic image of the anastomosis region after the application of fibrin glue

The endoscope was removed, and a catheter was inserted in the drain, which was used to insert two-component tissue glue in the fistula region, comprising a solution of sticky proteins, including fibrinogen and thrombin solution. As a result, the fistula was filled with glue, with partial release of the glue to the gastrointestinal lumen. See Figure 4.

On subsequent days, the drainage decreased until it stopped. Twenty days after the last procedure, the patient was discharged home with a full oral diet, with no thoracic drain. Currently, three months after discharge from the surgery department, the patient is in good general condition and continues perioperative chemotherapy.

## Discussion

Oesophagojejunostomy leakage after gastric resection remains a significant clinical problem. Basic management involves early diagnosis, the introduction of antibiotic therapy, parenteral nutrition, and drainage of the abscess in the anastomosis area. The purpose of such an initial procedure is to stabilize the patient and limit septic complications. With light leakage, conservative treatment may be fully successful, but in most patients, it is necessary to perform surgical procedures [5]. Reoperation with the creation of new anastomosis is a procedure carrying a high risk of mortality. Moreover, due to tissue fragility, present inflammation, and, frequently, poor general condition of the patient, there is a high risk of repeated leakage. The creation of a salivary fistula on the neck is a very invasive treatment and should only be used as a last resort [6].

Numerous reports present positive effects of endoscopic therapies, including the implantation of covered stents, use of endoscopic clips, endoscopic negative pressure therapy, and application of tissue glues [5-17]. Kim et al. assessed the effects of endoscopic treatment (the use of clips, glue, or stent implantation) in 33 patients with oesophagojejunostomy leakage. Such treatment was effective in 73% of patients with leakage smaller than 2 cm and only in 14% of patients with a larger fistula opening [5]. Schubert et al. reported good outcomes of endoscopic treatment in 25 out of 27 patients with leakage in the thoracic area. On this basis, they presented a procedure algorithm depending on the size of leakage. In patients with leakage below 30% of the circumference, they first proposed treatment with tissue glue and endoscopic clips and, if that was unsuccessful, stent implantation, whereas, for leakages covering 30-70% of the circumference, they proposed stent implantation as the initial treatment [7].

Endoscopic clips inserted through a working channel could be used to close small leakages. Unfortunately, such clips are small and offer only light pressure; therefore, they may be used for leakages detected early, when the wall is still elastic. With higher leakages, a technique based on clips and an endoloop may be used. This technique is possible with endoscopes with two working channels. An endoloop is positioned around the leakage, and it is fastened with clips to the leakage edges; then, closing the loop, the edges are brought together [5,8]. Another technique used for the closure of larger leakages is the use of over-the-scope clips (OTSCs); these are clips placed on a special endoscopic cap. After the endoscope insertion, the defect is drawn into the cap by suction, and a clip is released. One can also use forceps or an endoscopic hook in order to aspirate the anastomotic mucosa into the endoscopic cap. This method is highly successful in iatrogenic perforations, where even 20 mm defects may be closed. Unfortunately, their use for leakages and fistulas is still limited. Immediately after the procedure, the efficacy reaches 80%, but it falls to approximately 30% during a follow-up of 30 days. This is undoubtedly associated with an inflammatory process that stiffens the anastomotic wall [9].

Another technique used in the treatment of anastomotic leakage in the upper gastrointestinal tract is the implantation of fully or partially covered, self-expandable oesophageal stents. First, a guidewire is introduced under visual inspection, then, along the guidewire, a stent is positioned under radiological control. Such treatment may be successful even in 73%. The main drawbacks of this treatment include the need to use two or even three stents if the leakage persists, stent migration, and complaints of pain, which are often present for a few days after implantation [10-11].

Another method of treatment is based on the use of endoscopic vacuum-assisted closure (EVAC). This method involves inserting a polyurethane sponge combined with a vacuum system. There are two methods of sponge implantation. One method involves placing the sponge into the cavity formed as a result of anastomotic leakage, whereas the other one, used with smaller defects, involves inserting the sponge in the gastrointestinal lumen in such a way to make it adjacent to the leak. The negative pressure applied is usually in the range of 100-125 mmHg, and the sponges are replaced every three-four days [12]. The treatment efficacy of defects in the upper gastrointestinal tract ranges between 70% and 100% [12-13]. In a study comparing treatment with self-expandable stents and EVAC, healing was achieved in all EVAC patients, with 73% healing in the group treated with stents. Moreover, in the EVAC group, larger defects were treated, even such over 3 cm in diameter [11].

The next method used in the treatment of leakage involves the use of fibrin glue, consisting of two components containing fibrinogen and high thrombin concentration. During the application, the components are combined, followed by polymerization and the formation of a cross-linked form of fibrin. The best effect is achieved for small leakages and fistulas with a narrow channel [14], which is also confirmed by our observations. The fibrin glue may be administered in submucosal injections around the anastomosis leakage [5] or into the fistula channel [15-16]. The method of the glue application depends on the fistula location and on the technical aspects of its administration. Single glue application, as in the presented case, may be sufficient, but in some cases, may require a repeated application of the procedure for the fistula to be treated. The efficacy of fibrin glue in the treatment of upper gastrointestinal fistulas is about 57% [17].

## Conclusions

The complementary use of fibrin glue may be effective in the treatment of small oesophagojejunostomy leakages when other endoscopic methods are not sufficient.
